# X Inactivation and Escape: Epigenetic and Structural Features

**DOI:** 10.3389/fcell.2019.00219

**Published:** 2019-10-01

**Authors:** He Fang, Christine M. Disteche, Joel B. Berletch

**Affiliations:** ^1^Department of Pathology, University of Washington, Seattle, WA, United States; ^2^Department of Medicine, University of Washington, Seattle, WA, United States

**Keywords:** X chromosome, X inactivation, 3D-structure, LncRNAs, escape gene, epigenetics, dosage

## Abstract

X inactivation represents a complex multi-layer epigenetic mechanism that profoundly modifies chromatin composition and structure of one X chromosome in females. The heterochromatic inactive X chromosome adopts a unique 3D bipartite structure and a location close to the nuclear periphery or the nucleolus. X-linked lncRNA loci and their transcripts play important roles in the recruitment of proteins that catalyze chromatin and DNA modifications for silencing, as well as in the control of chromatin condensation and location of the inactive X chromosome. A subset of genes escapes X inactivation, raising questions about mechanisms that preserve their expression despite being embedded within heterochromatin. Escape gene expression differs between males and females, which can lead to physiological sex differences. We review recent studies that emphasize challenges in understanding the role of lncRNAs in the control of epigenetic modifications, structural features and nuclear positioning of the inactive X chromosome. Second, we highlight new findings about the distribution of genes that escape X inactivation based on single cell studies, and discuss the roles of escape genes in eliciting sex differences in health and disease.

## Introduction

Evolution of the mammalian sex chromosomes from a pair of autosomes resulted in the emergence of distinct heteromorphic chromosomes that govern sex determination (Graves, 2016). The Y chromosome contains few genes (∼70) and is present only in males, while the X chromosome contains many genes (∼900–1500) and is present in two copies in females and one copy in males. This contributes to gene dosage imbalance between X-linked and autosomal genes and between the sexes (Disteche, 2016). To relieve these imbalances two mechanisms of dosage compensation evolved: X upregulation of expressed genes in males and females, and X inactivation or silencing of one X chromosome in females (Deng et al., 2014).

Here we focus on X chromosome inactivation (XCI), a mechanism that results in silencing of a randomly chosen X chromosome in early female embryogenesis (Lyon, 1961). XCI is characterized by a cascade of molecular events beginning shortly after embryo implantation, and is faithfully maintained throughout somatic cells in an organism, providing a robust model to study epigenetic and structural changes associated with gene silencing (Galupa and Heard, 2018). This complex process starts with the *cis-*coating of the future inactive X chromosome (Xi) by the long-non-coding RNA (lncRNA) *Xist* (Borsani et al., 1991; Brockdorff et al., 1991; Brown et al., 1991). Layers of chromatin and DNA modifications catalyzed by proteins initially recruited by *Xist* RNA are then put in place over several days during early development for stable transcriptional silencing of each gene on the Xi (Froberg et al., 2013; Mira-Bontenbal and Gribnau, 2016). These modifications are associated with profound changes in the 3D structure and location of the Xi, both processes depending on X-linked lncRNA loci. The Xi adopts a bipartite structure consisting of two superdomains of chromatin condensation separated by the lncRNA locus *Dxz4*, and the Xi visits the nucleolus, a process facilitated by the lncRNA *Firre* (Zhang et al., 2007; Rao et al., 2014; Deng et al., 2015; Minajigi et al., 2015; Yang et al., 2015; Giorgetti et al., 2016; Fang et al., 2019).

Despite the multiple layers of gene repression that stabilize XCI, a subset of developmentally critical genes remains expressed, albeit at a lower level, from the Xi (Carrel and Willard, 2005; Berletch et al., 2011). These escape genes adopt chromatin signatures and structural features more akin to those found in regions of active transcription (Balaton and Brown, 2016). Such genes can have higher expression in females, leading to sex differences in normal physiology and in susceptibility to disease. Abnormal escape gene dosage contributes to a milieu of deleterious phenotypes including infertility, intellectual disability, immune diseases, and cancer (Disteche, 2016; Balaton et al., 2018).

This review focuses first on mechanisms that govern X chromosome structure and nuclear location in relation to XCI, with a specific emphasis on the role of X-linked lncRNAs in these processes. We next discuss mechanisms that allow a select subset of genes to escape silencing in the context of the repressed Xi environment and how single-cell RNA sequencing has been used to identify novel escape genes. Lastly, we review new data on the role of escape gene dosage in sex differences in health and disease.

## Long Non-Coding Rnas Control Epigenetic and Structural Features of the Inactive X Chromosome

The importance of lncRNAs in controlling nuclear structure and gene expression has become increasingly clear (Engreitz et al., 2016). Here we consider three X-linked lncRNAs, *Xist*, *Dxz4*, and *Firre*, which have been implicated in various aspects of the onset and maintenance of XCI.

### Xist

Recent reviews have considered the role of *Xist* in great detail (Mira-Bontenbal and Gribnau, 2016; Galupa and Heard, 2018). Thus, we will focus our discussion on issues related to structural changes on the Xi. One of those is a localized chromatin conformation change at the X inactivation center (XIC), which is essential for the correct initiation of XCI. The XIC harbors both the *Xist* locus and its antisense transcription unit *Tsix*, together with multiple other loci that regulate *Xist* (Froberg et al., 2013; Galupa and Heard, 2018). The *Xist* and *Tsix* promoters lie in separate but adjacent regions of local chromatin interactions called topologically associated domains (TADs) (Nora et al., 2012). Interestingly, swapping the *Xist/Tsix* transcriptional unit and placing their promoters in each other’s TADs leads to a switch in their expression dynamics, indicating the topological partitioning of the XIC is critical for proper initiation of XCI (van Bemmel et al., 2019).

**FIGURE 1:**
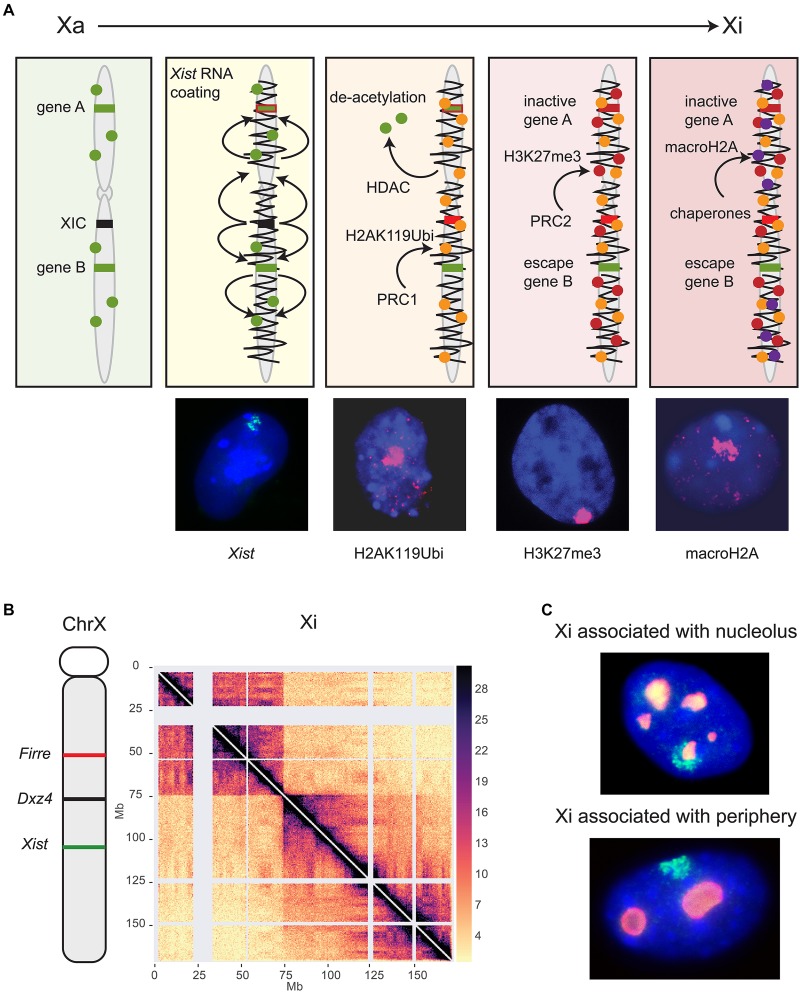
Long non-coding RNAs control epigenetic and structural characteristics of the Xi. **(A)** XCI begins with the expression of *Xist* located in the XIC (black). *Xist* RNA spreads (squiggly black line) along the X chromosome and recruits several protein complexes (see text). Major histone modifications take place, including histone deacetylation by HDAC3, followed by ubiquitination of H2AK119 mediated by the PRC1 complex, methylation of H3K27 mediated by the PRC2 complex, and finally incorporation of histone macroH2A. Gene A represents a gene that becomes inactivated (red) while gene B is an escape gene (green) that remains unchanged. This schematic focuses on histone modifications and does not show recruitment of other proteins or other repressive epigenetic modifications such as DNA methylation. Shown below the schematic are examples of female mouse nuclei showing an *Xist* cloud (green) after RNA-FISH, and enrichment of histone modifications (pink) by immunostaining of H2AK119Ubi, H3K27me3, and macroH2A. Nuclei are counterstained by Hoechst 33342. **(B)** The genomic location of *Firre*, *Dxz4* and *Xist* is indicated on a schematic of the mouse X chromosome along a Hi-C contact map of the Xi in mouse Patski cells. Two superdomains of frequent contacts are separated by the *Dxz4* region. The color scale shows normalized contact counts [adapted from a published figure (Bonora et al., 2018) in Nature Communications, under Springer Nature Publishing License: http://creativecommons.org/licenses/by/4.0/. **(C)** The Xi preferred locations are near the nucleolus or the nuclear periphery, as shown in examples of mouse fibroblast nuclei after RNA-FISH for *Xist* (green) to locate the Xi and immunostaining for nucleophosmin (red) to locate the nucleolus. Nuclei are counterstained by Hoechst 33342.

Once XCI is initiated, chromosome-wide structural changes give rise to the condensed Barr body coated by *Xist* RNA (Figure 1). Imaging studies show the rapid formation of a nuclear compartment devoid of transcriptional machinery and euchromatic marks, in which X-linked genes, initially located at the periphery of the *Xist* RNA cloud, adopt a more internal position when silenced (Chaumeil et al., 2006; Clemson et al., 2006). Subsequent chromatin conformation analyses by Hi-C demonstrate that *Xist* is essential for the formation of the unique Xi bipartite structure further discussed below (see section “*Dxz4*”) (Minajigi et al., 2015; Giorgetti et al., 2016). Most of the Xi shows attenuation of local TADs and of large A/B compartments of active and inactive chromatin, normally evident on autosomes and the active X chromosome (Lieberman-Aiden et al., 2009; Dixon et al., 2012; Minajigi et al., 2015; Giorgetti et al., 2016). *Xist* RNA pull-down studies have identified two RNA-binding proteins implicated in liquid-to-solid phase transition, FUS and hnRNPA2, suggesting the possibility that phase separation facilitates heterochromatin formation and Xi silencing (Calabrese et al., 2012; Patel et al., 2015; Ryan et al., 2018). Indeed, most proteins within the *Xist* interactome are predicted to be prone to phase separation. By high-resolution RNA-FISH about one hundred *Xist* foci can be identified on the Xi, these foci having a comparable shape, size, and morphology to other phase-separated condensates such as paraspeckles and stress granules (Cerase et al., 2019).

*Xist* RNA-mediated gene silencing is accomplished through recruitment of proteins that establish epigenetic and structural modifications on the Xi (Figure 1A; Chu et al., 2015; McHugh et al., 2015; Minajigi et al., 2015; Moindrot et al., 2015; Monfort et al., 2015). Originally, spreading of *Xist* RNA and its protein complexes was thought to be linear, but new studies have revealed complex interactions between epigenetic and genomic features, such as genomic distance from the *Xist* locus, gene density, and proximity to long interspersed nuclear elements (LINE) that act as waystations to enhance *Xist* RNA coating throughout the Xi 3D space (Sousa et al., 2019). Sequence analysis of *Xist* RNA reveals that a significant proportion of the primary RNA sequence is comprised of blocks of local tandem repeats (named A-F) with different functions in XCI. The utmost importance of the A-repeat in X silencing but not in *Xist* RNA coating was recognized early on, and has been further strengthened by demonstrating that it recruits the transcriptional repressor SPEN (Wutz et al., 2002; Zhao et al., 2008; Nesterova et al., 2019). The B-repeat together with a short part of the C-repeat are crucial for spreading of *Xist* RNA and for attracting the polycomb silencing complexes PRC1 and PRC2 (Pintacuda et al., 2017; Nesterova et al., 2019). Conversely, ablating PRC1 or PRC2 impairs *Xist* spreading, supporting interactive roles for this mega-protein-RNA complex (Colognori et al., 2019).

A new study has now clarified the precise order of appearance of histone modifications relative to X silencing (Żylicz et al., 2019). Importantly, loss of histone acetylation, in particular H3K27ac, is clearly one of the very first events following *Xist* RNA accumulation during XCI initiation (Figure 1A). Indeed, the histone deacetylase HDAC3 is pre-loaded at putative enhancers and is vital for efficient silencing of most genes on the Xi. Ubiquitination of histone H2K119 is then initiated by the nuclear matrix-PRC1 protein complex (hnRNPK-PCGF3/5-PRC1), signaling subsequent recruitment of other PRC1 complexes and of PRC2 (Figures 1A,B; Pintacuda et al., 2017). Tri-methylation of histone H3K27 is mediated by PRC2 and appears slightly later, even after gene silencing (Żylicz et al., 2019). The chromatin scaffolding protein SMCHD1 (structural maintenance of chromosomes flexible hinge domain-containing protein 1) plays an important role in gene silencing and structure of the Xi (Blewitt et al., 2008). A recent study proposes the Xi bipartite structure forms via an intermediate condensation step mediated by SMCHD1, in which A/B compartments initially fuse into S1/S2 compartments that subsequently merge into the compartment-less architecture of the Xi (Wang et al., 2018). Consistent with this finding, SMCHD1 loss of function results in the appearance of sub-megabase domains, A/B compartments, and a partial restoration of TAD boundaries on the Xi (Gdula et al., 2019). These changes are associated with de-repression of some X-inactivated genes and a local decrease in H3K27me3, but this reactivation is not observed in immortalized mouse embryonic fibroblasts, suggesting SMCHD1 facilitates H3K27me3 enrichment during XCI (Sakakibara et al., 2018; Gdula et al., 2019). At other heterochromatic regions of the genome SMCHD1 co-localizes with the repressive histone mark, H3K9me3, a process mediated by LRIF1 (Ligand Dependent Nuclear Receptor Interacting Factor 1). However, SMCHD1 remains enriched over the Xi even when SMCHD1-LRIF1 interactions are perturbed, suggesting an alternative mechanism by which SMCHD1 is targeted to the Xi, possibly via histone H2AK119 ubiquitination (Brideau et al., 2015; Jansz et al., 2018). Two later events that lock in silencing of the Xi are replacement of histone H2A by macrohistone H2A and DNA methylation of CpG islands by DNMT3B (Figure 1A; Gartler and Riggs, 1983; Costanzi and Pehrson, 1998; Gendrel et al., 2012).

### Dxz4

During the establishment of XCI the Xi condenses in two superdomains of long-range contacts separated by a region that contains the conserved *Dxz4* lncRNA microsatellite repeat (Figure 1B; Deng et al., 2015; Minajigi et al., 2015; Darrow et al., 2016; Giorgetti et al., 2016). This bipartite configuration is present in both mouse and human, albeit with different superdomain sizes, and deletion of *Dxz4* specifically from the Xi results in disruption of the bipartite structure in both species, indicating a conserved function (Darrow et al., 2016; Giorgetti et al., 2016; Bonora et al., 2018). The deleted Xi acquires a configuration that resembles the Xa with enhanced TADs and compartments, but only in part, suggesting that, in addition to *Dxz4*, other factors control the Xi configuration.

CTCF-mediated interactions between the *Dxz4/DXZ4* loci and other X-linked loci appear to be integral to forming chromatin loops for packaging the Xi. Indeed, *Dxz4*/*DXZ4* bind the zinc-finger protein CTCF and components of the ring-shaped cohesin complex only on the Xi (Horakova et al., 2012a, b). Elsewhere in the genome convergent CTCF binding motifs at the base of a chromatin loop clearly favor strong interactions, and the inversion of CTCF sites disrupts loop formation (de Wit et al., 2015). The mouse *Dxz4* locus contains a bank of CTCF motifs arranged in tandem orientation, while the human locus contains two banks of motifs with a different orientation (Horakova et al., 2012a, b). Our group reported that inversion of the mouse *Dxz4* locus results in a massive reversal in long-range contacts, indicating that the *Dxz4* locus itself acts as a structural platform for frequent long-range contacts with multiple X-linked loci in a direction dictated by the orientation of its CTCF motifs (Bonora et al., 2018). The anchoring of megabase size chromatin loops at *Dxz4* causes the appearance of a line (or flame) emanating from *Dxz4* in the contact map (Figure 1B). Whether contacts between *Dxz4* and other X-linked loci rapidly fluctuate in individual cells remain to be determined. Surprisingly, deletion of *Dxz4* causes only minor reactivation of X-linked genes and few changes in escape gene expression (Giorgetti et al., 2016; Bonora et al., 2018). A more recent study reported no changes in gene expression at all, suggesting inconsistencies between cell lines (Froberg et al., 2018). Furthermore, mice with a deletion of *Dxz4* on the Xi show no apparent phenotype (Andergassen et al., 2019). Thus, the bipartite structure of the Xi has no clear function in gene regulation of the Xi at this point. However, conservation of the locus and of the bipartite structure between human and mouse suggest preservation of function.

### Firre

*Firre* is another X-linked lncRNA locus that influences the epigenetic features and 3D structure of the Xi. We and others have shown that *Firre* is transcribed only from the active X chromosome (Calabrese et al., 2012; Froberg et al., 2018; Andergassen et al., 2019; Fang et al., 2019). Multiple isoforms of the *Firre* transcripts including lncRNAs and circular RNAs have been reported (Izuogu et al., 2018). Like *Dxz4*, the *Firre* region harbors many local repeats including the R0 repeat that recruits the chromatin organizers CTCF and YY1, as well as RAD21, a component of the cohesin ring complex (Yang et al., 2015; Hacisuleyman et al., 2016). The *Firre* locus recruits the nuclear matrix protein hnRNPU and interacts with many genomic regions, which might explain why depletion of *Firre* RNA causes widespread autosomal gene dysregulation (Hacisuleyman et al., 2014; Andergassen et al., 2019; Fang et al., 2019; Lewandowski et al., 2019). In regards to XCI, depletion of *Firre* RNA in differentiated fibroblasts does not disrupt *Xist* coating or gene silencing; however, a loss of H3K27me3 is observed on the Xi (Yang et al., 2015; Fang et al., 2019). In contrast, embryonic stem cells depleted of *Firre* prior to differentiation show no changes in H3K27me3 (Froberg et al., 2018). Together, these results support a role for *Firre* RNA in maintenance but not initiation of H3K27me3 enrichment on the Xi in differentiated cells.

*Firre* RNA is also important for maintenance of Xi location within the nucleus. Indeed, depletion of *Firre* in differentiated cells causes a decrease in perinucleolar and nuclear periphery association of the Xi (Yang et al., 2015; Fang et al., 2019). The perinucleolar and periphery compartments are often associated with heterochromatin and nucleolar location of the Xi has been proposed to be important for faithful replication of its epigenetic state (Figure 1C; Zhang et al., 2007; de Wit and van Steensel, 2009). Association of the Xi with the lamina may be facilitated by the lamin B receptor (LBR) recruited by *Xist* RNA (Chen C.K. et al., 2016). However, both the active and inactive X chromosomes are preferentially located near the nuclear periphery, suggesting that factors unrelated to XCI may control positioning (Bischoff et al., 1993). Surprisingly, tethering of one or both XIC alleles to the nuclear lamina via a TetR-EGFP-LaminB1 fusion protein does not disrupt XCI initiation, which would imply that XIC pairing and a visit to the nucleolus are not essential for XCI initiation (Pollex and Heard, 2019).

Deletion of *Firre* on the Xi does not disrupt the bipartite organization of the Xi, but causes localized changes in contact distribution, which may reflect disruption of the long-range contacts between *Firre* and *Dxz4* thought to secure Xi compaction (Darrow et al., 2016; Barutcu et al., 2018; Bonora et al., 2018; Froberg et al., 2018; Fang et al., 2019). Long-range contacts between loci may help isolate the Xi in a specific compartment or phase of the nucleus either near the nuclear periphery or the nucleolus. The lncRNA loci *Xist*, *Firre*, and *Dxz4* could play a concerted role in condensation and isolation of the Xi in a specific phase, which would ensure differential regulation of the two X chromosomes. Few studies have directly tested such a hypothesis due to the challenges in experimental design (Heard et al., 2004).

## Detection of Genes That Escape X Inactivation by Single-Cell Analyses

**FIGURE 2:**
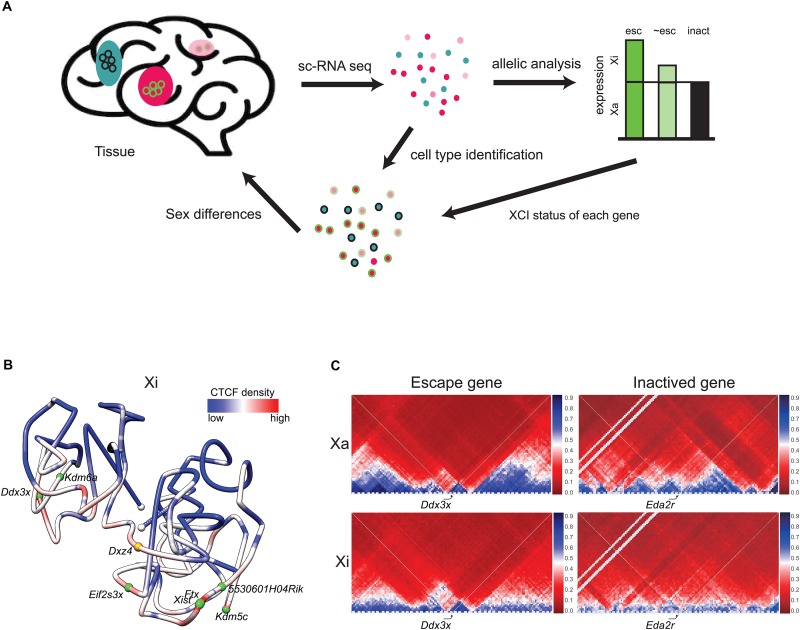
Escape genes distribution and structure. **(A)** Single-cell RNA-seq (scRNA-seq) enables determination of patterns of escape from XCI in cell types within a tissue. Beginning with a tissue, for example brain, individual cell types (colored pink, light pink, and blue) can be identified by scRNA-seq. Subsequent SNP analysis determines the escape status of each X-linked gene based on reads from the Xa and the Xi (escape gene, esc, colored green; inactivated gene, inact, colored black; gene that partially escapes, ∼esc, colored light green). Each cell type can be associated with the escape status of an ensemble of X-linked genes. For example, a specific cell type (pink) shows escape of the example gene (circled green), while another cell type (blue) shows inactivation of this same gene (circled black). Combination of data on an ensemble of genes can potentially inform sex differences in a particular cell type. **(B)** 3D model of the Xi at 1 Mb resolution in mouse brain colored to display the density of allelic CTCF binding (red indicates more binding and blue, less binding). Two domains of condensation are seen separated by *Dxz4* at the hinge. The white dots indicate chromosome ends, the orange dot, *Dxz4*, and the green dots, escape genes. Escape genes tend to be located at the outside of the Xi 3D structure [adapted from a published figure (Deng et al., 2015) in Genome Biology, under Springer Nature Publishing License: http://creativecommons.org/licenses/by/4.0/]. **(C)** Partial Hi-C contact maps (4 Mb resolution) of the active (Xa) and inactive (Xi) X chromosomes in a 4 Mb region around the escape gene *Ddx3x* and the inactivated gene *Eda2r* highlight the attenuation of TADs (blue) on the mouse Xi, except at the escape gene *Ddx3x* where TADs are visible on both the Xi and Xa. The color scale shows normalized contact counts (blue, higher contact count, red, lower contact count) [adapted from a figure (Bonora et al., 2018) published in Nature Communications, under Springer Nature Publishing License: http://creativecommons.org/licenses/by/4.0/].

Despite being located in a constitutively repressed environment, a select subset of genes has evolved mechanisms to avoid silencing and thereby remain expressed from the Xi. A number of genes escape XCI in an individual-, tissue-, and cell type-specific manner, which can cause sex differences in gene expression (Berletch et al., 2015; Cotton et al., 2015; Tukiainen et al., 2017; Balaton et al., 2018). In mouse, 3–7% of X-linked genes escape transcriptional silencing, while that number increases to 20–30% in human (Berletch et al., 2015; Balaton and Brown, 2016). Comparisons between species show that a core set of genes escapes XCI in most cells and tissues in mammals, while other genes vary between cell types, tissues and species. New approaches by single-cell RNA sequencing (scRNA-seq) combined with SNP (single nucleotide polymorphism) analyses of allelic gene expression provides data on thousands of individual cells of varying types and thus promises a more complete picture of the variation in escape status between cell types (Figure 2A). Assigning escape status to a given gene formally requires finding biallelic reads in single cells or in a tissue with completely skewed XCI. A main advantage of single cell approaches is that tissues with random XCI can be analyzed, but allelic dropout can cause problems for genes with low expression (Reinius and Sandberg, 2015). A limited scRNA-seq analysis of about 1,000 cells representing two human cell types has uncovered genes (e.g., *FHL1* and *ATP6AP2*) with incomplete XCI in a subset of cells, and also confirmed heterogeneity of XCI for *TIMP1* (Tukiainen et al., 2017). Another allelic scRNA-seq in human fibroblasts shows little overlap with other studies in terms of identified escape genes, highlighting the difficulty of building a consensus (Wainer Katsir and Linial, 2019). Variability in escape from XCI between individuals has been confirmed in a single-cell allelic expression analysis from five individuals (Garieri et al., 2018).

Developmental scRNA-seq studies have followed XCI progression during embryogenesis. A study of human preimplantation embryos reports that bi-allelic X-linked gene expression may persist until the blastocyst stage, together with dampening of both alleles (Petropoulos et al., 2016). However, re-analysis of these and additional data has provided a more detailed panorama of random XCI from human oocyte to blastocyst, demonstrating progressive establishment of mono-allelic X-linked gene expression and of X upregulation to maintain balance of expression throughout the genome (Moreira de Mello et al., 2017; Zhou et al., 2019). Unfortunately, very little information on escape genes is included in these studies, despite findings of extensive sex differences in overall gene expression. In mouse, XCI dynamics during embryonic stem cell differentiation show a gradual decrease in expression of escape genes, consistent with partial spreading of silencing (Chen G. et al., 2016).

## Structural and Epigenetic Features of Escape Genes

Expectedly, genes expressed from the Xi lack epigenetic signatures characteristic of inactivated genes and appear to be located away from repressive genomic elements. A recent study using allele-specific PRO-seq and predictive machine learning shows that primary determinants of escape from XCI include distance from *Xist* and density of LINE elements (Sousa et al., 2019). Interestingly, escape genes often cluster in domains, a common finding in human, while mouse escape genes are often isolated (Tsuchiya et al., 2004; Prothero et al., 2009; Berletch et al., 2011). In general, escape regions lack repressive histone marks such as H3K27me3, and are enriched in active histone marks such as acetylation, and in transcription elongation marks including RNA PolII S2P and H3K36me3 (Figure 1A; Disteche and Berletch, 2015; Sousa et al., 2019). DNA hypomethylation of CpG islands is a reliable predictor of escape status, which has been successfully used to identify escape genes in an array of human tissues where allelic analyses are difficult due to few SNPs and/or absence of XCI skewing (Cotton et al., 2015; Duncan et al., 2018). Surprisingly, escape genes in brain and liver adopt specific DNA methylation signatures that include enrichment in non-CG hypermethylation (mCH) throughout their gene body, which may help maintain an open chromatin structure (Keown et al., 2017; Duncan et al., 2018).

Escape genes tend to reside toward the outside of the compacted inactivated interior of the Xi (Figure 2B; Chaumeil et al., 2006; Heard and Bickmore, 2007; Splinter et al., 2011; Deng et al., 2015; Bonora and Disteche, 2017). However, it remains to be determined exactly how the 3D structure of the Xi influences the propensity for escape from XCI. One factor may be repeat E of *Xist*, which is required for localization of ASH2L, a component of the histone methyltransferase that methylates H3K4 for increased expression (Yue et al., 2017). Other factors may be involved, for example, the lncRNAs often found near escape genes (Reinius et al., 2010). Escape genes often co-localize with clusters of CTCF binding and with TADs, suggesting local Xi compartmentalization (Figure 2C; Giorgetti et al., 2016; Bonora et al., 2018). However, deletion of *Dxz4* and loss of the bipartite structure of the Xi causes little or no disruption in escape gene expression (Giorgetti et al., 2016; Bonora et al., 2018; Froberg et al., 2018).

It is increasingly clear that intrinsic genetic escape elements act in *cis* to facilitate expression from the Xi (Balaton et al., 2018). We and others have proposed that spreading of silencing into the escape region or vice versa spreading of gene activity into a silenced gene may be prevented by insulator elements such as CTCF or YY1, but these elements may not be sufficient and mechanisms may differ between escape genes (Filippova et al., 2005; Li and Carrel, 2008; Horvath et al., 2013; Chen C.Y. et al., 2016). Interestingly, a BAC harboring the human escape gene *RPS4X* inserted at the silenced *Hprt* locus in mouse retains escape status *in vivo*, both through the onset of XCI and the life of the mouse (Peeters et al., 2018). It will be interesting to identify the apparently conserved insulator elements involved in this process and to test their role in shaping regions of escape, for example by isolating them into separate chromatin loops or phases within the nucleus.

## Role of X-Linked Genes in Sex Differences and in Disease

One main consequence of escape from XCI is differential gene expression between males and females (Mele et al., 2015). A recent study based on thousands of transcriptomes spanning 29 human tissues provides a detailed survey of sex-biased gene expression in humans and demonstrates that expression of escape genes is usually female-biased (Tukiainen et al., 2017). However, a subset of escape genes located in the pseudoautosomal region shared between the X and Y chromosomes is male-biased, probably due to lower expression in females due to spreading of silencing on the Xi (Tukiainen et al., 2017). The biological implications of these sex bias remain largely unexplored. While it is clear that certain escape genes, e.g., *Kdm6a*, and its Y-paralog *Uty* are expressed in different parts of the mouse brain, their role in phenotypic sex differences has not been clarified (Xu et al., 2008). In fact, few traits have been linked to sex bias in X-linked gene expression in normal healthy individuals. One example is longevity, with recent evidence suggesting that having two X chromosomes prolongs lifespan, independent of gonadal sex. This was demonstrated by using the four core genotype (FCG), a mouse model capable of differentiating the effects of hormones versus sex chromosome complement, which showed that XX mice with either ovaries or testes lived longer than XY mice of either gonadal phenotype (Davis et al., 2019). In addition, having two X chromosomes leads to improved blood pressure regulation and an increase in the capacity to blunt the effects of brain injuries (Pessoa et al., 2015; McCullough et al., 2016).

In terms of disease susceptibility there is ample evidence suggesting that sex bias in X-linked gene expression play a role. For example, having two X chromosomes increases the risk of developing autoimmunity [reviewed in Syrett and Anguera (2019)]. This phenomenon may be a result of unusual XCI patterns in immune cells, which could leave certain X-linked genes involved in immune response susceptible to reactivation (Wang et al., 2016; Syrett et al., 2019). This is supported by studies demonstrating that the Toll-like receptor seven gene, which escapes XCI in human lymphocytes, causes systemic lupus erythematosus when overexpressed in mouse models (Deane et al., 2007; Souyris et al., 2018). The X chromosome harbors a high number of genes important in brain function, and dosage of some escape genes has been implicated in neurological phenotypes such as seizures and Autism Spectrum Disorder (Shoubridge et al., 2019). For example, loss of function mutations in the escape gene, *IQSEC2*, contribute to the manifestation of phenotypes that include moderate to severe intellectual disability (Shoubridge et al., 2019). Another example is *KDM5C*, an escape gene that encodes a histone demethylase and regulates neuronal development and function (Iwase et al., 2016; Scandaglia et al., 2017; Kim et al., 2018). Mutations in *KDM5C* cause intellectual disability in males and females, illustrating the dosage sensitivity of this gene (Brookes et al., 2015).

Variability in expression of escape genes may contribute to sex differences in predisposition to certain cancers (Arnold and Disteche, 2018). Many types of cancers are sex-biased in nature and some of those skewed toward a male prevalence may be explained by mutations in X-linked escape genes called EXITS (Escape from X Inactivation Tumor Suppressor) (Table 1; Dunford et al., 2017). One example is bladder cancer, with incident rates ranging from three to five times higher in men than women (Edgren et al., 2012). Recent studies in FCG mice show that XX mice with bladder cancer survive at increased rates compared to XY mice regardless of gonadal sex, suggesting that X-linked gene dosage is an intrinsic determinant of survival (Kaneko and Li, 2018). Interestingly, the female-biased escape gene *KDM6A* is a strong tumor suppressor that acts through demethylation-dependent and -independent mechanisms to reduce bladder cancer cell proliferation (Kaneko and Li, 2018).

**TABLE 1:** Human non-PAR escape genes as tumor suppressors in male-biased cancers.

**Gene^∗^**	**Escapes status^¶^**	**Cancer type**	**Male to female ratio**	**References**
*KDM6A*	Ubiquitous	Bladder	3-5:1	Kaneko and Li, 2018
		Lymphoma	1.6:1; 4:1	Morton et al., 2006; Horesh and Horowitz, 2014
		Glioblastoma	2:1	Sun et al., 2015
		T-cell acute lymphocytic leukemia	3:1	Goldberg et al., 2003; Van der Meulen et al., 2015
*KDM5C*	Ubiquitous	Clear cell kidney	2:1	Ricketts and Linehan, 2015
*ATRX*	Variable	Glioblastoma	2:1	Sun et al., 2015
*DDX3X*	Ubiquitous	Medulloblastoma	2:1	Sun et al., 2015; Valentin-Vega et al., 2016
		T-cell acute lymphocytic leukemia	3:1	Goldberg et al., 2003; Valentin-Vega et al., 2016

*^∗^Gene list based on (Dunford et al., 2017). ^¶^Escape status as reported in Patrat et al. (2009) and Cotton et al. (2013, 2015).*

Disorders of sex chromosome number such as Turner syndrome (45,X) and Klinefelter syndrome (47,XXY) directly implicate escape genes in abnormal phenotypes, since such genes would be haplo-insufficient and overexpressed, respectively, in these conditions. One of the hallmark of Turner syndrome is premature ovarian failure and infertility. The X chromosome is three-fold more enriched for genes expressed in female reproductive organs when compared to autosomes, indicating a role in female fertility (Liu, 2019). Defining the contribution of specific X-linked genes in abnormal Turner phenotypes is a work in progress. For example, the escape genes *KDM6A* and *TIMP1* are hypothesized to be involved in premature ovarian failure and aortic aneurysm formation, respectively (Trolle et al., 2016; Viuff et al., 2019). Recent screens of X-linked copy number variation (CNV) in cohorts of healthy women and those with primary ovarian insufficiency (POI) show a high prevalence of deletions encompassing escape genes as well as lncRNAs (Yatsenko et al., 2019). Of course, escape genes would not be the only X-linked genes involved in Turner infertility, since all X-linked genes become reactivated in female primordial germ cells, a process mediated by PR-domain containing protein 14 (PRDM14) for removal of H3K27me3 on the Xi (Mallol et al., 2019). The infertility observed in Klinefelter individuals could potentially also result from abnormal escape gene dosage, but another large factor would be abnormal meiotic pairing of the sex chromosomes. Interestingly, any abnormal X chromosome numbers (XXY, XXX or X) cause a general disruption of DNA methylation patterns at autosomal genes, demonstrating widespread epigenetic effects of X aneuploidy (Trolle et al., 2016; Skakkebaek et al., 2018). Furthermore, the number of X and/or Y chromosomes influences spatial chromosome conformation, particularly of the active X chromosome, but the role of this structural change is not elucidated (Jowhar et al., 2018). Together, these findings implicate improper X-linked gene dosage as a causative factor in disease phenotypes ranging from brain function, cancer susceptibility, to impaired fertility.

## Perspective

While a great deal has been learned about the various lncRNAs and proteins that control structural and epigenetic features of the Xi and its silencing, their exact modes of action remain to be further studied. In terms of the 3D structure of the Xi it will be of great interest to define factors involved in nuclear compartmentalization and phase separation. Additional experiments are needed in order to link the specific 3D structure and nuclear location of the Xi with its distinct epigenetic landscape. Moreover, little is known about contacts between each X chromosome with the rest of the genome in female cells and tissues, and about such contacts with the heterochromatic Y chromosome in male cells and tissues. Tissue-specific differences in the arrangement of chromosomes within the nucleus are poorly understood, and few functional studies have examined the consequences of manipulating chromosomal location. The epigenetic controls of escape from XCI also warrant further functional studies. Unfortunately, current analyses toward identifying escape genes in specific cell types and tissues are limited due to the relatively small number of informative polymorphisms in human. However, single-cell analyses are progressing at a fast rate, with some methods allowing analyses in thousands of cells in tissues to establish maps of gene expression or accessibility in a whole organism, as shown for example in a recently published atlas of mouse tissues/cell types (Cusanovich et al., 2018). While there is compelling evidence of sex differences in susceptibility to disease, understanding the role of individual sex-linked genes will require careful manipulation of their dosage.

## Author Contributions

JB, HF, and CD outlined and wrote the review. All authors approved the final manuscript.

## Conflict of Interest

The authors declare that the research was conducted in the absence of any commercial or financial relationships that could be construed as a potential conflict of interest.
